# A novel multi-enzyme preparation produced from *Aspergillus niger* using biodegradable waste: a possible option to combat heterogeneous biofilms

**DOI:** 10.1186/s13568-020-00970-3

**Published:** 2020-02-21

**Authors:** Arashdeep Kaur, Valbha Rishi, Sanjeev Kumar Soni, Praveen Rishi

**Affiliations:** 1 Department of Microbiology, Panjab University, Chandigarh, India; 2 Department of Civil Engineering, National Institute of Technical Teachers’ Training and Research, Chandigarh, India

**Keywords:** *Aspergillus niger*, Biofilm, Carbohydrases, Enzyme cocktail, Extracellular polymeric substance (EPS)

## Abstract

Extracellular polymeric substance (EPS) produced by the microorganisms provides protection and stability to them when they are encased within biofilms. Heterogeneous polysaccharides form a major constituent of the EPS and are crucial for the formation and integrity of the biofilms**/**slime. Thus, breakdown of polysaccharides might help in dispersion of biofilms from abiotic surfaces. In the present study we isolated a fungus, *Aspergillus niger* APS, capable of concurrently producing a cocktail of carbohydrases and optimized the conditions for higher yields of all the enzymes by one variable at a time (OVAT) approach. The optimization studies resulted in 1.5 to 12 fold augmentation in the enzyme yields using biodegradable waste. Further, keeping in view the heterogeneous nature of polysaccharides in biofilm matrix, the in-house produced enzyme cocktail was used for the dispersal of biofilms formed by *Salmonella enterica* serovar Typhi, *Escherichia coli* and *Staphylococcus aureus*. Treatment with enzyme preparation caused 90.23 ± 4.0, 82.64 ± 5.0 and 76.32 ± 5.0% reduction of the biofilms formed by these organisms respectively which was also evidenced by Field emission scanning electron microscopy (FESEM) revealing the loss of biofilm architecture. Interestingly, the enzyme cocktail could also remove viscous slime formed under natural conditions in the kitchen drainage pipe (KDP). To the best of our knowledge, this is the first report on biotreatment of abiotic surfaces for removal of biofilms/slime formed under natural conditions. The study thus indicates the prospects of using multiple carbohydrases as an anti-biofouling agent on abiotic surfaces like equipments as well as implants/prostheses and pipelines.

## Introduction

Enzymes, also known as bio-catalysts, play an important role in the metabolism and other biochemical pathways in all living organisms including microorganisms, plants, and animals. Some of these bio-catalysts are of special interest and are exploited commercially for economical synthesis of numerous products on an industrial scale. Eco-friendly nature, low energy input and cost-effectiveness of enzymes, as compared to their chemical counterparts, make them a better candidate for various processes (Nigam 2013). As compared to other sources like plants and animals, the production of enzymes from microorganisms is considered more economical, easier and faster than the plant and animal cells. Further, the enzyme production by microorganisms is neither influenced by climatic or seasonal changes, nor by regulatory or ethical issues. Easy downstream processing of microbial extracellular enzymes further makes them a superior choice (Tufvesson et al. 2010). For these reasons, most of the enzymes used in various industries are obtained from microbial sources.

Although a number of microorganisms have been isolated and studied for the production of different enzymes, however, looking at the huge natural biodiversity, bioprospecting helps in tapping the ecosystem for the isolation of novel sources of enzymes and other metabolites. A number of industries require cocktail of enzymes rather than a single enzyme e.g. biofuel production by hydrolysis of lignocellulosic biomass (Rana et al. 2013; Binod et al. 2019), biohydrogen production (Ye et al. 2009), bio-deinking (Singh et al. 2019), to enhance digestibility and nutrient availability of animal feed (Alsersy et al. 2015) and detergent industry (Naganthran et al. 2017). Considering the economic point, a single organism capable of elaborating multiple enzymes is preferred for such applications. In this regard, fungi are preferred over other microorganisms, since they produce a broad range of enzymes (Chugh et al. 2016). Additionally, cost-effective production using cheap substrates is an important prerequisite for industrial use/commercialization. In view of these concerns, we made attempts to design an economically viable medium for the concurrent production of multiple enzymes from a selected fungal isolate after executing the desired optimization, as the latter becomes mandatory before exploiting any agents for its applied value. The multiple enzymes so produced, were evaluated for their potential as anti-biofouling agent to disperse the biofilms of pathogenic bacteria as well as to clean the slime formed on drainage pipe which is considered to be a matrix having various substrates.

Biofilm formation is one of the most remarkable survival strategies adopted by bacteria, in natural and in clinical settings (Flemming et al. 2016). From human teeth to industrial sites, biofilms can form wherever there are sufficient water and nutrients (Costerton et al. 1995). One of the major concerns associated with microbial biofilm formation is, biofouling, a complex interaction between the membrane material, dissolved substances, fluid flow parameters and microbial biofilms. For example, thick layers of slime found on the inner surface of drainage pipes, commonly known as ‘black gunk’ (containing human wastes, food residues, oils, soap films, hair, decomposing materials) are a focal point for heterogeneous biofilms formed as a result of intra and inter-species interactions among the microorganisms. Within biofilms, the bacteria are encased within extracellular polymeric substances (EPS) which protects them from antimicrobial agents and also provides structural integrity to biofilms (Payne and Boles 2016; Wang et al. 2018; Zhang et al. 2019). The EPS consists of various biopolymers, out of which heterogeneous polysaccharides are one of the predominant components. Strategies making use of enzymes including individual (Loiselle and Anderson 2003, Nijland et al. 2010; Kalpana et al. 2012; Lamppa and Griswold 2013**)** as well as mixture of enzymes (Orgaz et al. 2006; Orgaz et al. 2007; Singh et al. 2015; Fleming, et al. 2017) have been investigated to combat biofilms. Lequette et al. (2010) analyzed the cleaning efficiency of different polysaccharidases and proteolytic enzymes against bacterial biofilms commonly found in the food industry processing lines. Efficacy of proteases and carbohydrases like cellulases, amylase and pectinase have also been evaluated against the biofilms of *Pseudomonas* sp., *Bacillus subtilis* and *Staphylococcus aureus* (Orgaz et al. 2007; Singh et al. 2015) which these organisms may form in the clinical settings as well as on medical devices. *Therefore, keeping in view (i) the heterogeneity of the extracellular polysaccharides on the biofouled surfaces* and, *(ii) the cost incurred in procuring the purified enzyme preparations, a mixture of enzymes may be more beneficial for sufficient disruption of bacterial biofilms and clearing of slime*. This study thus envisages the perspective of developing a cost-effective and eco-friendly process to obtain multiple carbohydrases, capable of disrupting biofilms formed in vitro as well as the slime formed on kitchen drainage pipe (KDP) under natural conditions.

## Materials and methods

### Isolation of the fungal strain

A natural variant of the fungal strain belonging to genus *Aspergillus niger* was used in the present study. The strain capable of producing the required enzyme cocktail was isolated from the soil after screening various samples in the form of rotten foods, decomposed kitchen waste, decaying agricultural residues and soil collected aseptically from various places in and around Chandigarh, India. The isolates were screened for production of extracellular enzymes namely cellulases, hemicellulases, pectinase, amylases and alginate lyase enzymes by substrate hydrolysis method.

### Identification of the strain

The strain was identified to the genus level on the basis of macroscopic and microscopic features while the complete taxonomic status was established by 18S ribosomal sequencing taking the services of Eurofins Genomics, India. The phylogenetic tree was generated using the Maximum Likelihood method (Kimura 1980).

### Selection of suitable fermentation technique for the production of enzyme cocktail

To find out the choicest method for the production of an extracellular cocktail of carbohydrases from the isolated strain; surface, submerged and solid-state fermentation methods were tried. For surface and submerged fermentation, the media contained wheat bran (2%), yeast extract (0.25%) and peptone (0.25%). For solid-state fermentation 5 g wheat bran (50%), yeast extract (0.25%) and peptone (0.25%) moistened with 5 ml distilled water was used. These media were sterilized by autoclaving at 15 psi for 20 min, inoculated with five discs cut from the periphery of actively growing culture of fungal strain *Aspergillus niger* APS grown on potato dextrose agar (PDA) plates and incubated for 4 days. For submerged fermentation, incubation was done under shaking conditions (150 rpm) at 30 °C while for surface and solid-state fermentation, incubation was done under static conditions at 30 °C. After incubation, 100 ml distilled water was added to the flask of solid-state fermentation and kept for shaking for one hour at 30 °C. The contents of all the flasks were filtered through a sieve. Filtrates so obtained were centrifuged at 10,000 rpm at 4 °C for 15 min and the clear supernatants were used as the sources of extracellular enzymes.

### Enzyme assays

Cellulase (CMCase, FPase and β-1,4-glucosidase), hemicellulase (xylanase and mannanase), pectinase and glucoamylase activities in cell free supernatants were assayed by quantification of reducing sugars liberated by these enzymes from respective substrates followed by estimation by the 3,5-dinitrosalicylic acid (DNS) method (Miller 1959). The activities of enzymes were expressed in International Units (IU) where one unit each of the CMCase, FPase, β-glucosidase, xylanase, mannanase, pectinase and glucoamylase is equivalent to the enzyme that releases one µmole of end product in one min under standard assay conditions. α-amylase assay was performed by the method of Fuwa (1954) wherein one IU is equivalent to the amount of enzyme which reduces the color of starch-iodine complex by 10% in 10 min. In the case of alginate lyase, one unit has been defined as an increase in optical density (235 nm) of 0.010 per min (Sawabe et al. 1992).

### Time course studies for the production of multiple carbohydrases from the selected fungus

The time course for the production of all the carbohydrases i.e. cellulases, hemicellulases, pectinase, amylases, and alginate lyase, on wheat bran was studied by preparing different sets of 250 ml Erlenmeyer flasks and carrying out the surface fermentation under static conditions and submerged fermentation for 10 days at 30 °C. The production profiles of all the carbohydrases was studied by withdrawing flasks, in duplicate, at regular intervals of 24 h and the enzyme yields were determined as discussed above.

### Standardization of cultural and environmental conditions for optimization of co-production of various enzymes under surface culture fermentation

This study was attempted by assessing the effect of various carbon, nitrogen and mineral sources in the production medium as well as the effect of incubation temperature and pH of the medium in surface culture fermentation under static conditions for 6 days at 30 °C, unless otherwise stated. The working volume of the fermentation was 50 ml and fermentation was carried out in 250 ml Erlenmeyer flask.

### Effect of carbon sources

The effect of various carbon sources was studied by replacing wheat bran in the production medium with either of the cellulose, carboxymethyl cellulose, guar gum, pectin, starch, cellobiose, lactose, glucose, galactose, salicin, xylose at a concentration of 1% while concentration of de-oiled rice bran and kitchen waste (dried in hot air oven at 70 °C and crushed to powder form) was kept 2.0%. Medium containing wheat bran was used as control.

### Effect of various metal ions

Effect of various metal ions was studied by supplementing the medium with different metal salts including CaSO_4_, ZnCl_2_, K_2_HPO_4_, FeSO_4_, MnSO_4_, MgSO_4_, KH_2_PO_4_, and KCl, separately, at a concentration of 0.02%. Medium without supplementation of any metal salt was used as control.

### Effect of nitrogen source

The effect of nitrogen was studied by replacing peptone and yeast extract with various inorganic nitrogen sources including (NH_4_)_2_SO_4_, NH_4_Cl, NaNO_3_ and organic nitrogen sources including urea, yeast extract, mycological peptone, tryptone and corn steep liquor (CSL), keeping the final concentration of all the sources at 0.5% w/v. Medium without supplementation of any nitrogen source was used as control.

### Effect of incubation temperature

The effect of temperature was studied by incubating the flasks at different temperatures including 20°, 25° 30°, 35°, 40° and 50 °C

### Effect of initial pH of the medium

This was studied by adjusting the initial pH of the production medium at 2.0, 3.0, 4.0, 5.0, 6.0, 7.0, 8.0, 9.0 and 10.0 by the addition of HCl (1 N) or NaOH (1 N).

### Bacterial strains for biofilm formation

The bacteria used for biofilm production were *Salmonella enterica* serovar Typhi Ty2 (*S*. Typhi) (initially procured from Central Research Institute, Kasauli, India), *Staphylococcus aureus* ATCC 9144 and *Escherichia coli* MTCC 3222 (procured from IMTECH, Chandigarh, India).

### Quantification of biofilm formation by microtitre plate method

Quantification of biofilm formation was carried out in sterile polystyrene microplates by the method described by Stepanović et al. (2000). Briefly, the wells of a sterile polystyrene microplate were filled with 230 μl of medium. A quantity of 20 μl of overnight grown culture (cell count adjusted to 10^8^ CFU/ml) was added into each well. The plates were incubated for 24 h at 37 °C. The contents of the plate were poured off and washed three times with 250 μl of phosphate buffer saline (PBS; 0.01 M, pH 7.0). The biofilms were fixed with 250 μl of methanol per well. After 15 min, microplates were emptied and air-dried. Thereafter, microplates were stained with 250 μl per well of crystal violet for 10 min. Excess stain was rinsed off with sterile distilled water. The plates were air-dried, the dye bound to the adherent cells was resolubilized with 250 μl of 33% (v/v) glacial acetic acid per well and optical densities were read at 595 nm in a microplate reader. The negative control wells contained broth only.

### Standardization of biofilm formation

Biofilm formation by the selected pathogens was standardized in microtitre plates by varying the media (Luria broth (LB), brain heart infusion (BHI) broth and tryptic soy broth (TSB) for biofilm formation. The effect of incubation period on biofilm formation was also standardized by incubating and quantifying the biofilm formation after every 24 h for a period of 5 days.

### Biofilm removal efficiency of the enzyme cocktail produced by *A. niger* APS

To check the biofilms dispersal efficacy of the enzyme cocktail, the biofilms were developed on microtitre plates as described above. After biofilm development, the non-adherent cells were removed by washing with PBS. After washing, 250 μl of the enzyme preparation (filter sterilized) was added to the wells followed by incubation at 37 °C for 1 h. The in-house produced enzyme cocktail comprised multiple carbohydrases including CMCase (3.8 IU/ml), FPase (1.22 IU/ml), β-glucosidase (3.68 IU/ml), pectinase (14.2 IU/ml), xylanase (40 IU/ml), mannanase (7.4 IU/ml), glucoamylase (41.0 IU/ml), α-amylase (2760 IU/ml) and alginate lyase (3.3 IU/ml). Wells with biofilms and without any enzymatic treatment served as control. Heat-inactivated enzymes were also run in parallel. The biofilm removal efficiency of the enzyme cocktail was evaluated by percentage reduction method described by Pitts et al. (2003). Percentage reduction was calculated by the following formula:

$${\text{Percentage reduction}} = \left[ {\left( {{\text{C}} - {\text{B}}} \right) - \left( {{\text{T}} - {\text{B}}} \right)/\left( {{\text{C}} - {\text{B}}} \right)} \right] \times 100\%$$

where B denotes the average absorbance per well for blank (no biofilm, no treatment); C denotes the average absorbance per well for control wells (biofilms, no treatment); T denotes the average absorbance per well for treated wells (biofilm and treatment). The disruption of the biofilms was also checked by field emission scanning electron microscopy (FESEM).

### Field emission scanning electron microscopy (FESEM)

For FESEM, biofilms were grown by immersing the coverslips in the wells of a 6-well polystyrene plate. After biofilm formation, the coverslips were washed with phosphate buffer saline to remove the planktonic cells. To visualize the effect of enzymes, the biofilms developed on coverslips were incubated with the enzyme cocktail (filter sterilized) for 30 min. The control biofilms were incubated with PBS for 30 min. Thereafter, the biofilms on the coverslip were fixed with 2.5% glutaraldehyde for 1 h. The glutaraldehyde was washed off thrice with 0.1 M PBS (pH 7.2). After this, gradual dehydration was carried out by incubating the coverslips with increasing concentration of ethanol (30–90%), for about 15 min in each gradient. Final dehydration was done with 100% ethanol at room temperature and the dehydrated samples were dried, gold-sputtered and examined under Field Emission Scanning Electron Microscope (SU8010, Hitachi).

### Removal of slime formed on kitchen drainage pipe (KDP) under natural conditions

A piece of KDP with preformed thick slime layer was cut carefully. The piece was divided into four parts to be used as (i) control (no treatment) (ii) piece treated with phosphate buffer saline (0.05 M; pH 7.0) (iii) piece treated with enzyme cocktail and (iv) piece treated with heat inactivated enzymes. The effect of PBS, enzyme cocktail and heat inactivated enzyme cocktail was studied by immersing the KDP pieces in the respective solutions at room temperature. The effect was visualised after 2 h, 24 h and 48 h of incubation by visualizing the KDP pieces for the dispersal of slime layer, if any. The slime dispersed by the action of multiple carbohydrases was separated from KDP by washing with PBS and erosion of the slime was compared to other treatments.

### Statistical analysis

The data are expressed as mean ± standard deviation. Statistical significance between various groups was evaluated using one way analysis of variants (ANOVA) followed by Dunnett’s multiple comparison tests. In all data analysis, p-values of 0.001 or less (p ≤ 0.001) were considered significant.

### Accession number and availability of the strain

The isolated strain, designated as *Aspergillus niger* APS was deposited in general collection of Microbial Type Culture Collection (MTCC) under the accession number 12975.The 18S rRNA gene sequence (600 bp) of the strain was deposited in the GenBank with accession number MN559364.

## Results

### Isolation of the microorganism

After extensive screening of various samples, 38 isolates were selected during primary screening, out of which, 21 isolates were selected (on the basis of the clear zone size) and were subjected to secondary screening by growing them as submerged cultures on wheat bran based media (data not shown). A soil isolate S-34 capable of producing a cocktail of carbohydrases (cellulases, xylanase, mannanase, pectinase, amylases, and alginate lyase) in appreciable amounts, using wheat bran as substrate, was selected.

### Identification of the strain

Macroscopic and microscopic analysis: The isolated strain grew rapidly on Saboraud Dextrose Agar, the growth occurred as a mat which was initially white to yellowish in appearance and turned black as the conidia formed during further incubation. Microscopic examination performed using lactophenol cotton blue staining revealed septate and hyaline hyphae. Long conidiophores with brown to black conidia were also observed.

Molecular analysis: 18S rDNA sequencing of the isolated fungal strain S-34 revealed 600 bp sequence and was found to share high similarity with the species of genus *Aspergillus*. The ITS region sequence was used to carry out BLAST with the database of NCBI Genbank. Phylogenetic analysis revealed that the isolated strain was closely related to *Aspergillus niger* and was named as *A. niger* APS.

### Enzyme production by different fermentation techniques

Out of the different fermentation techniques tried, maximum enzyme productions were observed under surface fermentation followed by submerged fermentation and solid state fermentation respectively. Maximum CMCase (2.16 ± 0.03 IU/ml), FPase (0.284 ± 0.015 IU/ml), mannanase (3.96 ± 0.014 IU/ml), xylanase (42.83 ± 0.84 IU/ml), α-amylase (237.14 ± 7.12 IU/ml) and alginate lyase (2.03 ± 0.04 IU/ml) yields were achieved under surface fermentation. In contrast, pectinase and glucoamylase productivities were higher under submerged fermentation while β-glucosidase (2.6 ± 0.013 IU/ml) yields were optimal under solid state fermentation. Further, time course studies were performed under both the surface and submerged fermentations techniques in order to achieve maximal enzyme production.

### Time-course studies for co-production of various enzymes

**Fig. 1:**
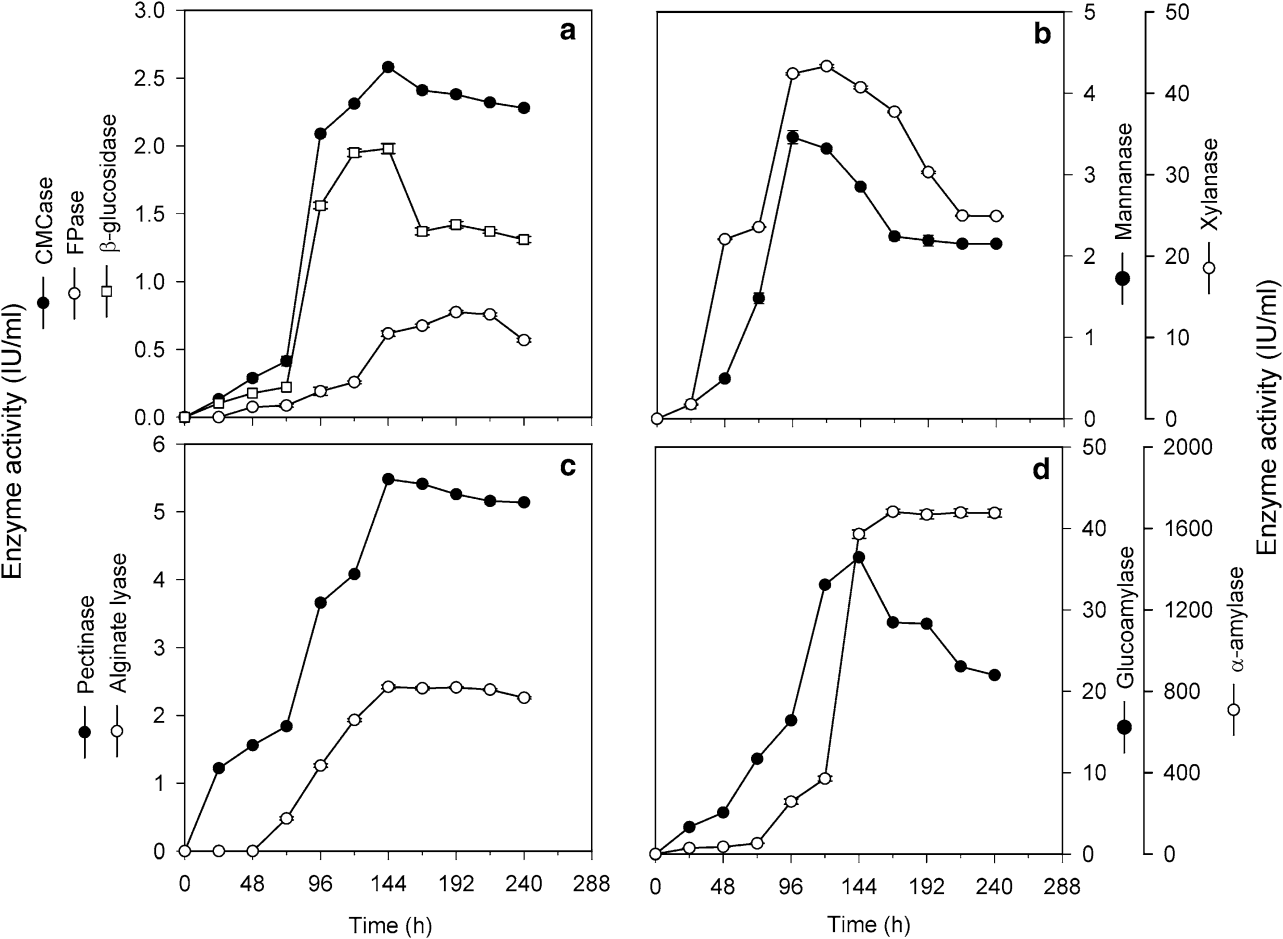
Time course for co-production of different enzymes **a** cellulases; **b** hemicellulases; **c** pectinase and alginate lyase; **d** amylases; over a period of 10 days (240 h) under surface fermentation

Production of most of the enzymes started as early as 24 h of incubation however, FPase and alginate lyase production started after 48 h and 72 h respectively (Fig. 1). Maximum CMCase (2.58 ± 0.027 IU/ml), β-glucosidase (1.98 ± 0.037 IU/ml), pectinase (5.48 ± 0.12 IU/ml), alginate lyase (2.42 ± 0.067 IU/ml) and glucoamylase (36.42 ± 0.28 IU/ml) activities were achieved after 6 days of incubation. On the other hand highest activities of mannanase (3.46 ± 0.082 IU/ml) and xylanase (43.33 ± 0.182 IU/ml) were achieved after 4 days while the maximum FPase (0.775 ± 0.013 IU/ml) and α-amylase (1681 ± 12.47 IU/ml) yields were achieved after 8 and 7 days of incubation respectively. Although different enzyme components were produced maximally at different incubation times, 6 days of incubation was chosen for further studies, as this time period gave appreciable yields of all the enzymes with least compromises. A similar trend was observed in submerged fermentation (Additional file 1: Fig. S1) wherein optimal enzyme activities were observed after different time intervals. However, in comparison to surface fermentation, maximal enzyme levels were achieved after a longer incubation period under submerged fermentation.

### Standardization of cultural and environmental conditions

#### Effect of carbon sources

Various carbon sources listed in Table 1 were selected to study their effect on co-production of various enzyme components in the cocktail. Maximum yields of FPase (1.24 ± 0.02 IU/ml), pectinase (8.52 ± 0.021 IU/ml), glucoamylase (42.67 ± 2.25 IU/ml), α-amylase (2651.6 ± 31.23 IU/ml) and alginate lyase (3.02 ± 0.014 IU/ml) were achieved when kitchen waste was used as the carbon source and maximum CMCase (2.27 ± 0.02 IU/ml) and xylanase (39.05 ± 0.44 IU/ml) productivities were observed with wheat bran. Highest β-glucosidase (1.77 ± 0.014 IU/ml) and mannanase (5.83 ± 0.021 IU/ml) activities were observed in presence of guargum. Since, wheat bran and kitchen waste were found to give optimal yields of the enzymes, therefore, these two carbon sources were further tried in combination and a further increment in the enzyme productivities was observed.

**Table 1:** Effect of various nutritional factors on co-production of various enzymes by *A. niger* APS

Nutritional factors Carbon Source	CMCase	FPase	β-Glucosidase	Pectinase	Mannanase	Xylanase	Gluco-amylase	α-Amylase	Alginate lyase
	Enzyme activity (IU/ml)								
De-oiled rice bran	1.76 ± 0.03*	0.687 ± 0.015*	0.535 ± 0.023	4.11 ± 0.07	2.31 ± 0.012	19.76 ± 0.105	27.43 ± 1.42	1228 ± 19.2	0.0
Kitchen waste	1.08 ± 0.01	1.24 ± 0.02	1.02 ± 0.02	8.52 ± 0.021	1.45 ± 0.012	19.04 ± 0.25	42.67 ± 2.25*	2652 ± 31.23	3.02 ± 0.014
Cellulose	1.68 ± 0.03	0.472 ± 0.02	0.228 ± 0.02	2.47 ± 0.015	0.0	0.0	28.26 ± 0.74	1160 ± 26.87	0.0
CMC	1.88 ± 0.04*	0.811 ± 0.02	0.647 ± 0.01	2.56 ± 0.03	2.3 ± 0.14	2.4 ± 0.081	31.23 ± 0.032	1748 ± 18.2	0.0
Guargum	1.72 ± 0.04	0.604 ± 0.01	1.77 ± 0.018	4.01 ± 0.017	5.83 ± 0.021	0.0	23.43 ± 0.76	1906 ± 24.56	0.87 ± 0.03
Pectin	1.70 ± 0.014	1.14 ± 0.014	0.849 ± 0.03	5.8 ± 0.012	4.5 ± 0.018	0.0	29.87 ± 1.06	1416 ± 21.46	0.834 ± 0.04
Cellobiose	1.37 ± 0.012	0.803 ± 0.02	0.122 ± .017	2.79 ± 0.024	0.43 ± 0.015	0.0	28.28 ± 2.26	1763 ± 28.26	0.962 ± 0.06
Lactose	1.40 ± 0.04	0.846 ± 0.02	0.080 ± 0.017	2.38 ± 0.014	0.152 ± 0.021	0.0	27.25 ± 1.12	1461 ± 23.52	1.26 ± 0.07
Glucose	1.32 ± 0.014	0.393 ± 0.02	0.251 ± 0.02	3.17 ± 0.017	0.501 ± 0.011	0.0	20.53 ± 0.98	2011 ± 38.26	2.34 ± 0.11*
Galactose	1.14 ± 0.04	0.219 ± 0.02	0.108 ± 0.014	2.32 ± 0.023	0.156 ± 0.012	0.0	27.48 ± 1.37	2139 ± 41.34	2.16 ± 0.08*
Starch	1.49 ± 0.015	0.994 ± 0.01	0.370 ± .011	3.67 ± 0.024	0.654 ± 0.018	1.43 ± 0.09	41.48 ± 3.26*	2659 ± 36.54	0.0
Salicin	0.89 ± 0.01	0.370 ± 0.05	0.108 ± 0.06	2.47 ± 0.013	0.0	0.0	4.56 ± 0.042	294 ± 6.43	0.0
Xylose	1.53 ± 0.032	0.678 ± 0.02*	0.939 ± 0.017	3.98 ± 0.014	0.686 ± 0.08	30.59 ± 1.24	18.42 ± 1.02	1288 ± 16.37	0.926 ± 0.03
Wheat bran + kitchen waste	2.61 ± 0.021	1.32 ± 0.013	2.48 ± 0.026	8.36 ± 0.024	3.42 ± 0.036	40.26 ± 0.47	41.33 ± 0.56	2632 ± 33.0	3.12 ± 0.04
Control (Wheat bran)	2.72 ± 0.02	0.683 ± 0.06	1.94 ± 0.03	5.54 ± 0.24	3.76 ± 0.01	39.05 ± 0.44	37.08 ± 0.42	1574 ± 2.50	2.16 ± 0.02
Metal ions									
CaSO_4_	2.94 ± 0.014	1.47 ± 0.017	2.89 ± 0.044^**#**^	10.31 ± 0.071^**#**^	4.73 ± 0.086	43.93 ± 1.02	44.02 ± 2.03	2643 ± 47	3.42 ± 0.046
MgSO_4_	2.75 ± 0.03	1.61 ± 0.022	2.26 ± 0.017	11.26 ± 0.34^**#**^	6.09 ± 0.11	46.27 ± 4.3^**#**^	43.27 ± 1.87	2620 ± 53	2.87 ± 0.011
K_2_HPO_4_	2.37 ± 0.01	0.949 ± 0.063	2.21 ± 0.026	9.72 ± 0.087^**#**^	5.88 ± 0.121	28.18 ± 1.26	44.05 ± 2.06	2094 ± 27^**#**^	2.63 ± 0.012
KH_2_PO_4_	2.41 ± 0.21	0.917 ± 0.04	2.75 ± 0.014	10.26 ± 0.124^**#**^	4.40 ± 0.98	33.85 ± 1.068	42.87 ± 1.17	2691 ± 38.65	2.54 ± 0.011
FeSO_4_	2.93 ± 0.022	1.10 ± 0.025	2.70 ± 0.11	8.31 ± 0.107	5.36 ± 1.03	40.17 ± 2.97	38.86 ± 2.37	2713 ± 29	1.87 ± 0.076^**#**^
MnSO_4_	2.10 ± 0.14^**#**^	0.931 ± 0.04^**#**^	2.17 ± 0.023	9.48 ± 0.98^**#**^	5.51 ± 1.21	29.5 ± 1.27^**#**^	35.54 ± 1.42	2285 ± 23^**#**^	1.56 ± 0.011^**#**^
ZnCl_2_	1.71 ± 0.023^**#**^	0.089 ± 0.02^**#**^	1.16 ± 0.07^**#**^	8.40 ± 0.031	2.35 ± 0.28	31.15 ± 0.243^**#**^	34.23 ± 0.86^**#**^	1987 ± 21^**#**^	1.42 ± 0.06^**#**^
KCl	1.95 ± 0.012^**#**^	0.618 ± 0.031^**#**^	2.40 ± 0.11	8.36 ± 0.067	4.95 ± 0.56	20.72 ± 0.87^**#**^	38.86 ± 1.57	2485 ± 35	2.89 ± 0.013
Control	2.64 ± 0.016	1.30 ± 0.03	2.46 ± 0.07	8.29 ± 0.083	3.37 ± 0.014	40.85 ± 1.63	41.26 ± 0.85	2612 ± 44	3.07 ± 0.016
Nitrogen sources									
Ammonium sulphate	2.07 ± 0.012	0.446 ± 0.03	2.20 ± 0.014*	11.5 ± 0.086*	3.18 ± 0.024	24.62 ± 2.03	33.04 ± 1.4*	2944.5 ± 22.58	1.83 ± 0.014
Sodium nitrate	2.64 ± 0.012*	0.920 ± 0.04	2.18 ± 0.012*	11.81 ± 0.065*	4.23 ± 0.042	25.63 ± 1.86	29.42 ± 1.26	1220 ± 12.36	1.27 ± 0.018
Ammonium chloride	1.57 ± 0.026	0.731 ± 0.03	1.96 ± 0.028	12.26 ± 0.102	3.08 ± 0.028	22.9 ± 1.74	35.49 ± 2.04*	3949 ± 56.75	0.86 ± 0.02
Urea	2.07 ± 0.014	0.589 ± 0.02	1.72 ± 0.016	7.98 ± 0.27	2.79 ± 0.014	24.25 ± 1.05	30.29 ± 2.85	2807 ± 23.37*	1.13 ± 0.017
Peptone	3.14 ± 0.060*	1.22 ± 0.01	2.25 ± 0.023*	11.04 ± 0.104	4.13 ± 0.033	37.25 ± 1.24	45.23 ± 1.22*	2692 ± 28.45*	2.13 ± 0.011
Yeast extract	2.83 ± 0.02*	0.993 ± 0.01	1.30 ± 0.067	8.53 ± 0.47*	1.80 ± 0.04	32.65 ± 0.68	37.75 ± 1.06*	2834 ± 18.78*	1.75 ± 0.02
CSL	3.79 ± 0.018	1.25 ± 0.012	3.76 ± 0.028	14.72 ± 0.56	7.72 ± 0.05	40.08 ± 2.02*	42.27 ± 2.84*	2740 ± 37.9*	3.17 ± 0.011*
Soyabean meal	1.65 ± 0.012	1.26 ± 0.016	2.07 ± 0.014*	11.09 ± 0.42	4.67 ± 0.058	22.65 ± 1.28	37.24 ± 1.06*	2932 ± 25.87	1.83 ± 0.012
Control	2.88 ± 0.035	1.56 ± 0.011	2.81 ± 0.022	10.86 ± 0.078	5.23 ± 0.034	42.23 ± 1.02	41.27 ± 2.03	2663 ± 31.66	3.26 ± 0.014

In case of carbon and nitrogen sources, all the values differ from the control significantly (p < 0.001) by Dunnett’s Multiple Comparison Test except those marked with*. In case of metal salts all the values marked with# differ from the control significantly (p < 0.001) by Dunnett’s Multiple Comparison Test

#### Effect of various metal ions

Table 1 shows the effect of supplementation of various metal ions on the enzyme yields. Out of the various metal ions used, CaSO_4_ and MgSO_4_ led to a significant increase in the activities of pectinase, mannanase and xylanase as compared to the control while there was no significant effect on the yields of cellulases, amylases and alginate lyase. In contrast, supplementation of MnSO_4_, ZnCl_2_ and KCl led to a significant decrease in the yields of cellulases, amylases, xylanase and alginate lyase.

#### Effect of various nitrogen sources

Various nitrogen sources including inorganic and organic (Table 1) were selected to study their effect on co-production of various enzyme components in the cocktail. Among the various inorganic and organic nitrogen sources tried, CSL was found to have significant effect on the yields of cellulases, pectinase and mannanase while no significant change in the yields of xylanase, glucoamylase and alginate lyase was observed. Maximum α-amylase yield was recorded with ammonium chloride followed by ammonium sulphate and soyabean meal. Other nitrogen sources led to a decrease in the yields of most of the enzymes in the cocktail. CSL was chosen as the nitrogen source for further studies as it led to an appreciable increase in the activities of enzymes with least compromises.

#### Effect of incubation temperature

As observed in Table 2 incubation temperature was found to have a profound effect on enzyme yields. No FPase, glucoamylase and alginate lyase activity was observed at 20 °C. However, production of all the enzymes was observed at 25 °C. Further rise in temperature led to an increase in the enzyme yields and maximum activities of all the enzymes in the cocktail was observed at 30 °C except xylanase, which was found to be maximum at 35 °C. Further rise in incubation temperature, lowered the enzyme yields.

**Table 2:** Effect of environmental factors on co-production of various enzymes by *A. niger* APS

Factor	CMCase	FPase	β-Glucosidase	Pectinase	Mannanase	Xylanase	Gluco-amylase	α-Amylase	Alginate lyase
	Enzyme activity (IU/ml)								
Incubation temperature (°C)									
20	1.44 ± 0.017	0.0	0.17 ± 0.04	4.5 ± 0.98	0.70 ± 0.003	11.33 ± 1.78	0.0	614.9 ± 5.58	0.0
25	2.45 ± 0.014	0.86 ± 0.024	0.84 ± 0.038	6.55 ± 0.106	2.08 ± 0.014	28.48 ± 0.31	18.42 ± 2.04	2392.3 ± 21.68	1.23 ± 0.04
30	3.84 ± 0.012	1.21 ± 0.04	3.62 ± 0.028	14.55 ± 0.15	7.33 ± 1.07	39.48 ± 1.32	42.31 ± 2.56	2774 ± 2.67	3.32 ± 0.016
35	2.26 ± 0.012	0.49 ± 0.026	1.95 ± 0.011	10.75 ± 0.304	3.04 ± 0.028	41.14 ± 1.04	41.87 ± 3.84	2073 ± 18.9	2.72 ± 0.016
40	2.37 ± 0.014	0.35 ± 0.06	1.85 ± 0.078	5.96 ± 0.098	2.39 ± 0.018	30.39 ± 1.86	36.42 ± 2.03	1182 ± 12.32	1.87 ± 0.011
50	0.37 ± 0.002	0.0	0.0	0.0	0.47 ± 0.01	0.0	0.0	0.0	0.0
Initial pH									
2.0	0.27 ± 0.06	0.31 ± 0.02	0.0	4.87 ± 0.068	0.38 ± 0.04	23.12 ± 2.14	0.0	433.3 ± 6.45	0.0
3.0	2.67 ± 0.014	0.53 ± 0.042	2.51 ± 0.012	8.35 ± 0.22	6.30 ± 1.08	31.04 ± 2.86	37.11 ± 3.14	2005.5 ± 23.25	1.26 ± 0.011
4.0	3.85 ± 0.014	1.24 ± 0.04	3.72 ± 0.20	13.92 ± 0.18	7.58 ± 0.98	41.58 ± 1.12	39.10 ± 4.48	2759.6 ± 30.62	3.26 ± 0.032
5.0	3.13 ± 0.012	1.02 ± 0.023	3.40 ± 0.012	14.26 ± 0.12	6.67 ± 0.23	41.82 ± 1.56	37.55 ± 2.28	2629.7 ± 35.56	3.53 ± 0.028
6.0	2.44 ± 0.014	0.98 ± 0.012	2.63 ± 0.025	13.34 ± 0.046	4.28 ± 0.067	42.05 ± 0.067	36.31 ± 1.13	2428.9 ± 27.45	4.48 ± 0.06
7.0	2.51 ± 0.018	0.92 ± 0.01	1.66 ± 0.015	6.14 ± 0.86	4.46 ± 0.42	48.41 ± 2.13	11.72 ± 1.86	2302.3 ± 20.45	4.73 ± 0.07
8.0	2.23 ± 0.014	0.89 ± 0.06	1.69 ± 0.011	5.67 ± 0.042	4.26 ± 0.045	48.02 ± 1.12	10.86 ± 1.12	2236.7 ± 24.37	3.52 ± 0.011
9.0	2.17 ± 0.016	0.77 ± 0.03	1.39 ± 0.012	5.37 ± 0.37	3.52 ± 0.014	44.84 ± 3.86	9.87 ± 0.09	2214.7 ± 36.78	1.72 ± 0.017
10.0	2.17 ± 0.011	0.73 ± 0.03	0.83 ± 0.011	4.51 ± 0.021	3.12 ± 0.012	42.43 ± 1.07	2.59 ± 0.15	2104.6 ± 27.42	1.12 ± 0.06

#### Effect of initial pH of media

To study the effect of pH on enzyme productivities of the cocktail, the initial pH of the media was varied from 2.0 to 10.0 and incubation was done at 30 °C for 6 days. Enzyme productivities were observed over a broad pH range. With an increase in pH from 2.0 to 4.0, the enzyme productivities also increased. Maximum CMCase, FPase, β-glucosidase, glucoamylase, mannanase and α-amylase productions were observed at pH 4.0 and maximum pectinase yield was at pH 5.0. Xylanase and alginate lyase activities were maximum at pH 7.0. The results are shown in Table 2.

#### Standardization of biofilm formation

Out of the different media used for biofilm standardization, LB and BHI were found to support maximum biofilm production by *E. coli* and *S.* Typhi respectively but there was no significant difference in biofilm produced with the use of these two media. In case of *S. aureus*, TSB was found to be the best for biofilm formation, respectively (Additional file 1: Fig. S2a). Further, maximum biofilm formation was observed after 72 h of incubation in case of *E. coli* and *S. aureus* while for *S*. Typhi maximum biofilm formation was observed after 48 h of incubation (Additional file 1: Fig. S2b) and further increase in incubation time led to a decrease in biofilm biomass.

### Biofilm removal efficiency of the enzyme cocktail

**Fig. 2:**
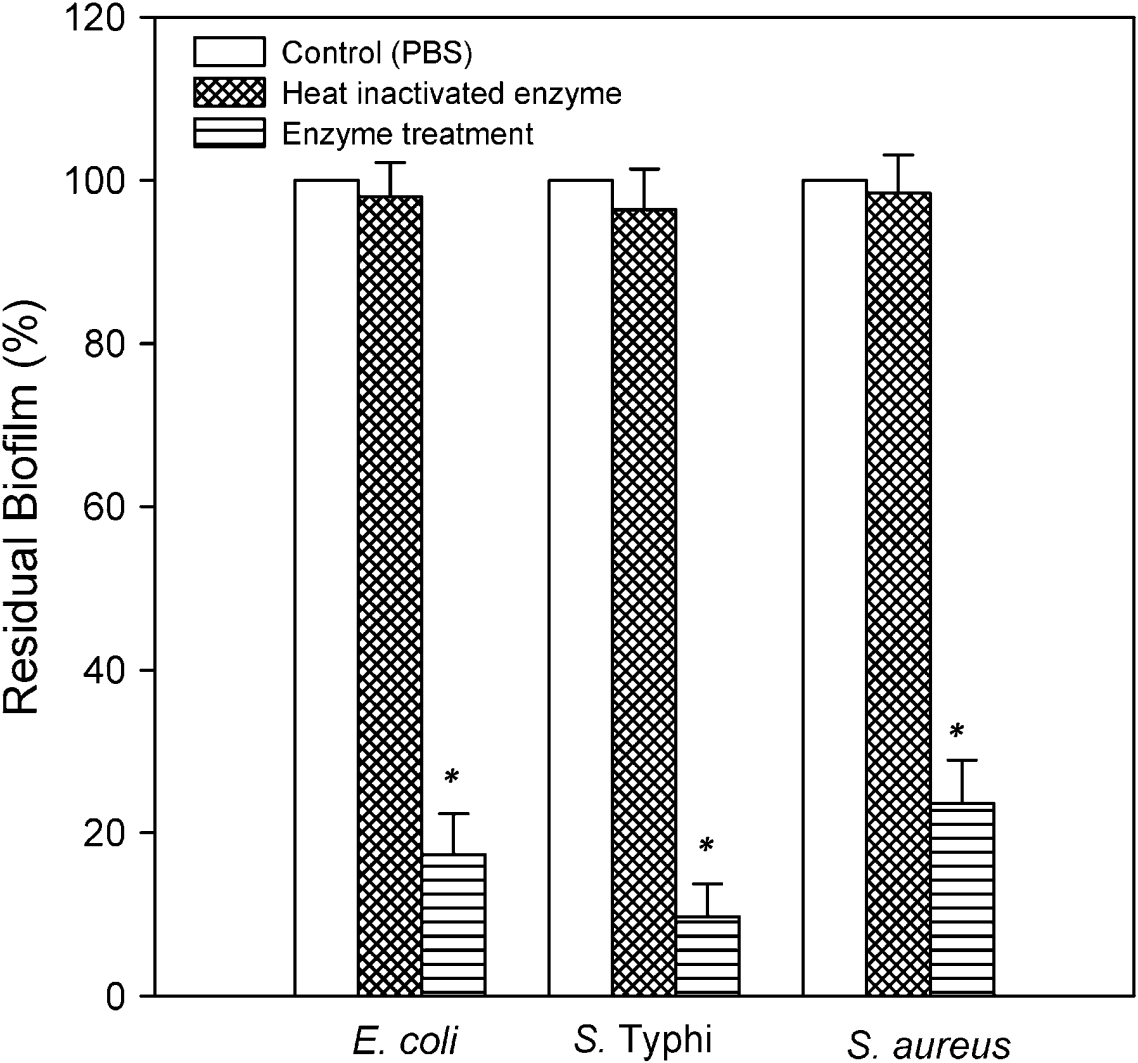
Residual biofilm (%) after treatment with in-house produced enzyme cocktail. * represents p < 0.0001 versus control

The enzyme cocktail produced by the fungal isolate was found to eradicate the biofilms of the selected pathogens. However, the enzyme cocktail was found to be more effective against the Gram-negative bacteria i.e. *E. coli* and *S*. Typhi wherein 82.64 ± 5.0% and 90.23 ± 4.0% eradication was observed respectively. In the case of *S. aureus* 76.32 ± 5.0% reduction was observed (Fig. 2).

### FESEM analysis

**Fig. 3:**
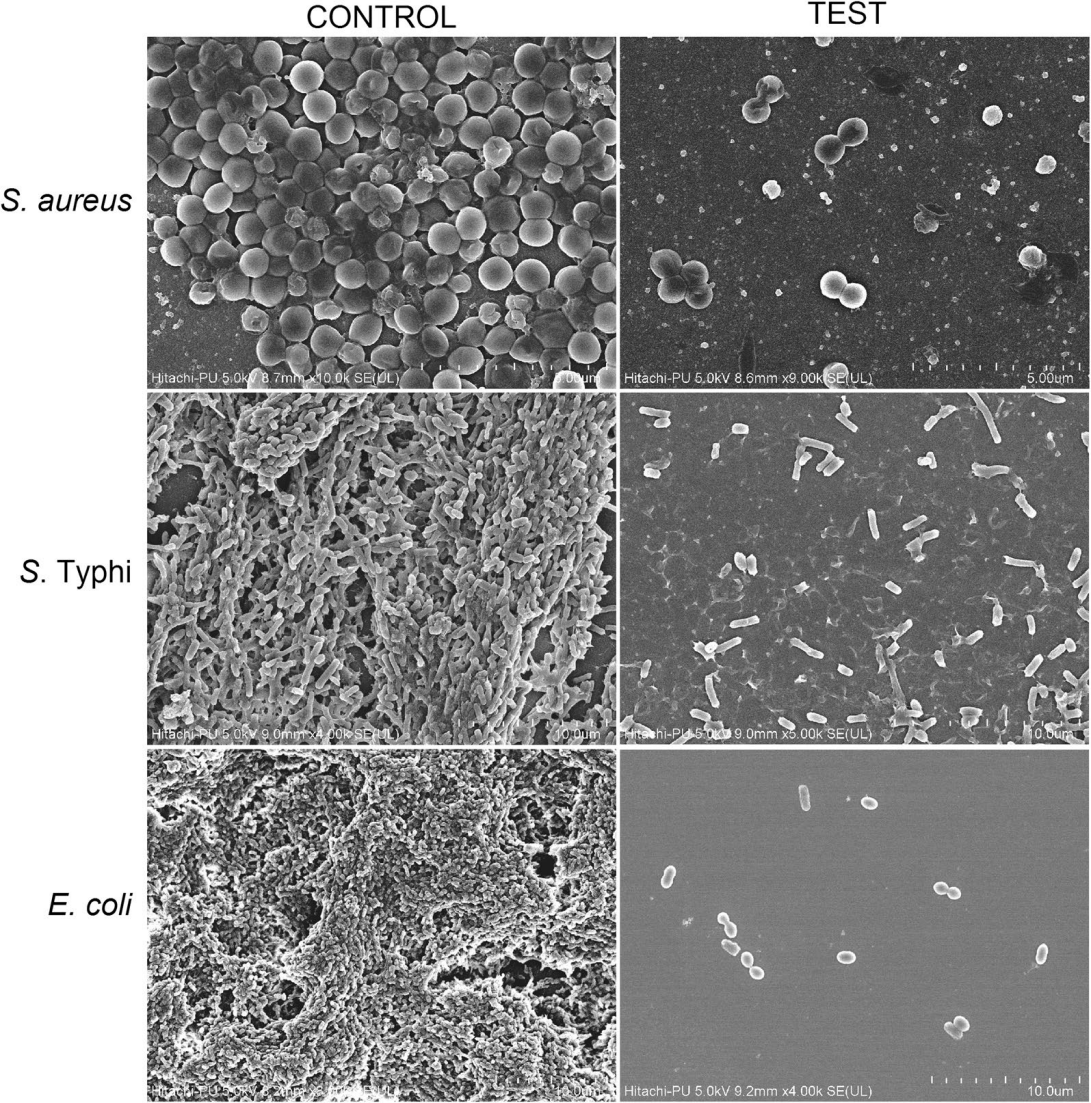
FE-SEM images of the control (untreated) biofilms established on coverslips and test biofilms (treated with in-house produced enzymes)

To further investigate our results, FESEM analysis of biofilms, both enzyme treated and untreated, was performed. As shown in the Fig. 3, intact biofilm architecture with confluent growth of the cells was observed in the control biofilm (untreated), while in case of the enzyme treated biofilms, the biofilm architecture was completely lost and significant disruption of the biofilms was observed. However, the morphology of the cells, after biofilm disruption, was not affected but the density of the cells was significantly reduced as compared to the control or untreated biofilms.

### Removal of slime from KDP

**Fig. 4:**
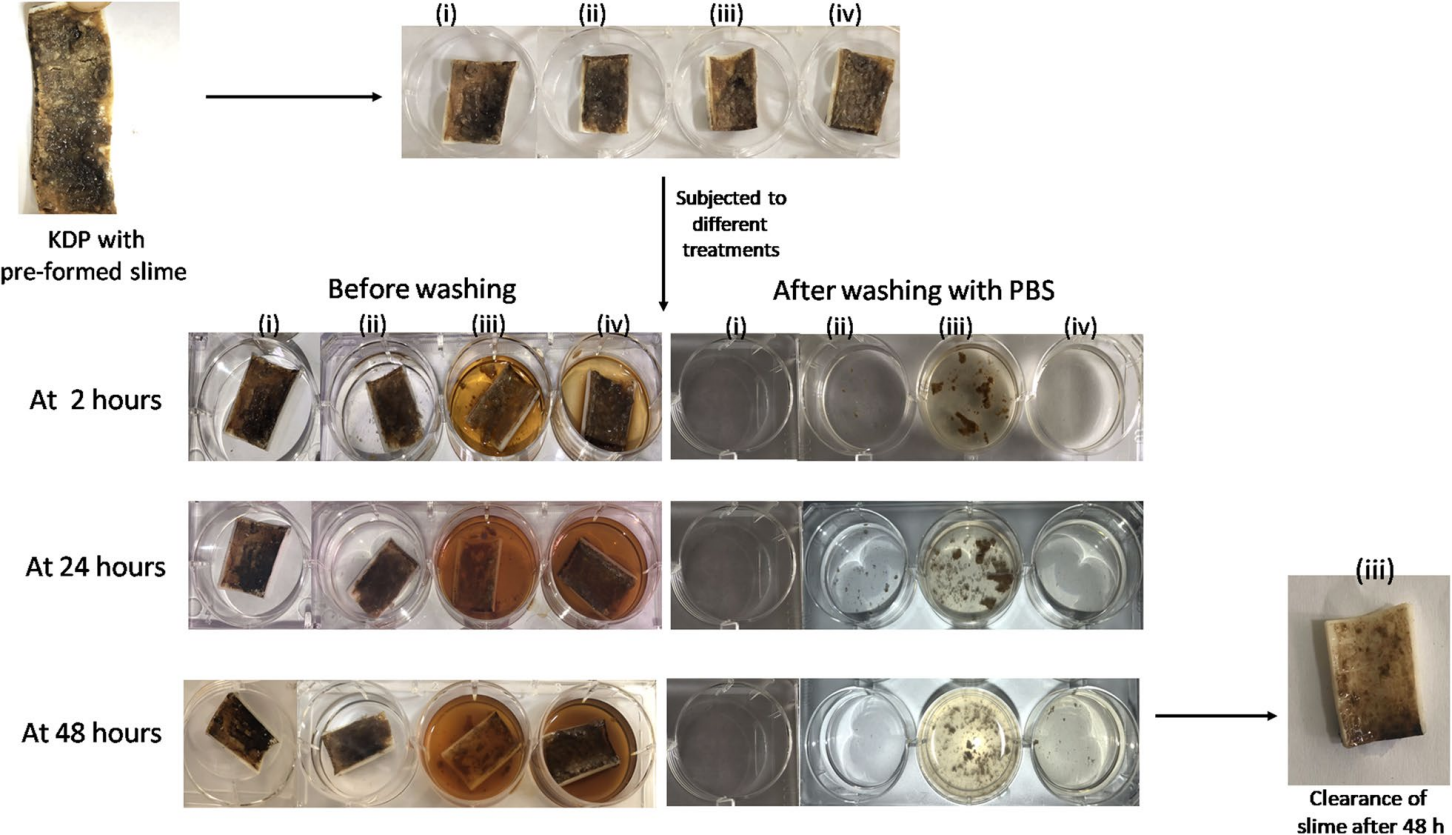
Clearance of pre-formed slime (Black gunk) deposited on kitchen drainage pipe (KDP) by the action of in-house produced enzyme cocktail after 2 h, 24 h and 48 h. The placement of 4 pieces cut from KDP is shown in the figure as (i) Control (Untreated) (ii) Treated with PBS (iii) Treated with enzyme cocktail (iv) Treated with heat-inactivated enzymes

The efficacy of the multiple carbohydrases was tested to degrade/disperse thick slime layer developed on the inner surface of KDP. The effect of the enzymes was clearly visible within initial 2 h wherein clear dispersion of the slime was observed (Fig. 4), while no effect was observed on slime present on the control KDP as well as those treated with PBS and heat inactivated enzyme preparation. Further clearance of the slime was observed after 24 h wherein, a significant difference as compared to the control, PBS treated and heat inactivated enzymes could be observed. The action of enzymes continued till 48 h and a marked clearance of the slime was observed as compared to the controls.

## Discussion

Looking at the heterogeneous nature of the polysaccharides present in the biofilms and the complexity of these polysaccharides due to inter-species and intra-species interactions of microorganisms in biofilms, a fungal strain capable of producing multiple carbohydrases including cellulases, hemicellulases, pectinase, amylases and alginate lyase was isolated. Following isolation, we tried to develop an economically viable medium for maximal co-production of all the enzymes.

After repeated screening, we were able to isolate a fungal strain S-34 that elaborated the required enzyme cocktail in appreciable amounts. The isolate was found to be closely related to *Aspergillus niger* and was named *A. niger* APS. This genus is considered as a model for enzyme production, as it is a source of various commercially important enzymes. Out of the different fermentation techniques tried for enzyme, surface fermentation was found to be the best. Since, the aim of the study was to develop an economically viable media for the co-production of enzymes, this was a favourable finding as surface fermentation is easy to implement and control as compared to submerged and solid state fermentation. The equipment required is simple and there is no need of aeration or agitation of the fermentation broth which makes the whole fermentation process economical, in addition to the easy recovery of the product ultimately (Shah et al. 1991).

Evaluation of time course studies is of paramount importance for the production of enzymes from fungi. In the present study, optimum levels of different enzymes were found to be produced at different time intervals. The highest levels of different enzymes were achieved between 4 to 8 days, beyond which a gradual decrease in the enzyme(s) level was observed which was probably due to change in the pH or due to release of proteases or some inhibitory metabolites during the stationary phase. In addition, repression effect due to the accumulation of products of various enzymatic reactions might also result in decreased enzyme production (Chugh et al. 2016). Earlier, maximum concurrent production of various carbohydrases has been reported between 4–6 days of incubation from *Aspergillus niger* using rice straw, wheat bran (Kang et al. 2004) and deoiled rice bran (Chugh et al. 2016) as substrate.

Production of carbohydrases is inducible and strongly influenced (induced or repressed) by the carbon sources. In the past, various lignocellulosic substrates including wheat bran, deoiled rice bran, rice straw, wheat straw, corn stover and sugar cane bagasse etc. have been screened for various enzyme productions (Singh et al. 2014; Imran et al. 2017). In the present study, presence of diverse carbohydrates both in the kitchen waste and wheat bran might have induced the production of various carbohydrases by the isolate *A. niger* APS). In addition, high water binding capacity and high porosity of wheat bran allows easy penetration of the fungal hyphae and has been reported to induce variety of carbohydrases (Palmarola-Adrados et al. 2005; Jain and Agrawal 2018). The cellobiohydrolase (CBH) system in fungi like *Aspergillus* has been reported to be induced by cellulose, xylose and lactose. On the contrary, CBH system has been reported to be repressed in the presence of the glucose and other easily metabolizable sugars due to carbon catabolite repression (CCR) effect. Alginate lyase production has also been reported to be influenced by the type of carbon source present in the environment (Fu et al. 2008). Likewise, production of other carbohydrases including hemicellulases, pectinase and amylases is also inducible and wheat bran has been indicated to be a good inducer of these enzymes (Teixeira et al. 2000; Martínez-Trujillo et al. 2009; Irfan et al. 2012; Singh et al. 2014; Tallapragada and Venkatesh 2011), thereby indicating that the fungus can sense the availability of various carbohydrates in the environment and make the endogenous alterations to utilize those by production of various carbohydrases for its own benefit. In the study, effect of different metal salts was also studied and different enzymes were found to be induced maximally by different metal ions which are essential for the growth of fungi as they act as cofactors for various enzymes. Metal ions like phosphorus and sulphur are important constituents of nucleic acid and amino acids respectively.

In addition to carbon sources and metal salts, nitrogen sources (both organic and inorganic) have been found to induce hydrolytic enzymes. In this study, CSL was found to have a profound effect on production of various carbohydrases. CSL (a rich source of soluble proteins and amino acids) consists of dry matter (50 ± 2.31%), crude proteins (40 ± 2.1%), ash (10 ± 0.43%), nitrogen free extract (16 ± 1.10%) and lactic acid (21 ± 1.22%) (Nisa et al. 2006). Various studies support the use of organic nitrogen sources like peptone, yeast extract, urea, casein, soyabean meal etc. for the production of carbohydrases (Gautam et al. 2011; Deswal et al. 2011). Improved pectinase yields have also been reported using ammonium salts (Galiotou-Panayotou et al. 1997; Fawole and Odunfa, 2003). Peptone, KNO_3_ and yeast extract have been found to stimulate alginate lyase production (Sugano et al. 1995; Fu et al. 2008). Role of supplements like nitrogen sources becomes very critical in production of multienzymes as not many supplements enhance simultaneous production of all enzymes in a single bioreactor. Studies on the effect of supplementation of different carbon and nitrogen supplements show that not all sources act as enhancers for simultaneous production of these enzymes in a single fermentation system (Negi and Banerjee 2010). In the present study, certain nitrogen sources might have modified the pH of the substrate which in turn influenced the growth of *A. niger* APS and therefore, the enzyme yields or some inhibitory component in the complex nitrogen sources might had resulted in the decreased enzyme yields. It has been reported that nitrogen sources like ammonium are taken up in antiport with one proton, due to which there is a substantial acidification of the medium (Bhanja et al. 2007). This acidification causes growth to stop and results in inactivation of enzymes.

Optimum production of all the enzymes in the cocktail was achieved at incubation temperature of 30 °C except xylanase, which was found to be maximum at 35 °C. The optimum temperature for carbohydrase production varies with the strain, however a range between 25 and 30 °C has been found to be optimum for various fungi (Ja’afaru and Fagade 2010; Gautam et al. 2011) and the optimum temperature for enzyme production depends on whether the culture is thermophilic or mesophilic. Enzyme production varies with the fungal growth and 30 °C has been reported to be optimum for growth of many fungi including *A. niger*. Higher temperatures (above 30 °C) have been found to cause cell death as the cell membrane composition is altered and protein catabolism is also stimulated (Bansal et al. 2012). Maximum amylase yields from *A. oryzae* were reported at an incubation temperature of 30 °C (Sivaramakrishnan et al. 2007; Puri et al. 2013). Apart from the factors discussed, there is also a critical relation between initial pH, fungal growth and enzyme production. pH plays a vital role in the structural modifications of the enzymes’ active site, glycosylation of the enzymes and transport of molecules and enzymes across the membrane. Although the optimal growth and enzyme production varies from species to species but in case of fungi, the optimal pH range lies from 3.0 to 5.5 (Puri et al. 2013; Chugh et al. 2016). In the present study, the optima for production of different enzymes varied and majority of the enzymes were found to be produced optimally at acidic pH. The results are in corroboration with earlier studies wherein high yields of cellulases from *Trichoderma reesei* Rut C-30, xylanase from *A. niger* and *T. viridae*, mannanase from *Sclerotium rolfsii* and *A. terreus* were achieved when pH of the medium was 4.0–4.5, 5.5, 5.0 and 6.0 (Großwindhager et al. 1999; Xiong et al. 2004; Liu et al. 2006; Soliman et al. 2012). Similar to our studies, neutral and alkaline pH have been found to favour alginate lyase production (Fu et al. 2008). Although, there are reports on multiple enzyme production by different fungi using submerged fermentation and solid state fermentation (Table 3), however, to the best of our knowledge this is the first study wherein production of cocktail of nine different enzymes from a single strain using surface fermentation has been reported and further, the activities of most of the enzymes are better than reported in the literature.

**Table 3:** Comparison of the yields of various enzymes under different fermentation techniques among different fungi

Organism	Type of fermentation	Enzyme activity	CMCase	FPase	β-glucosidase	Pectinase	Mannanase	Xylanase	β-xylosidase	α-amylase	Glucoamylase	Alginate Lyase	References
*A. niger* KK2	SSF	IU/gds	130.0	19.0	94.0	–	–	5070.0	176.0	–	–	–	Kang et al. (2004)
Co-cultures of *A. niger* and *T. viride*	SmF	IU/ml	2.79	1.75	4.66	–	–	189.7	–	–	–	–	Ikram-ul-Haq et al. (2006)
*P. echinulatum*	SmF	IU/ml	3.0	0.9	1.5	–	–	42.0	–	–	–	–	Martins et al. (2008)
*A. niger* CJ-5	SSF	IU/gds	56.4	17.0	42.8	126.0	53.2	990.0	–	31,500	488.8	–	Janveja et al. (2013)
*P. echinulatum*	SmF	IU/ml	7.2	1.5	4.0	–	–	30.5	–	–	–	–	Reis et al. (2015)
*A. niger* P-19	SSF	IU/gds	115.76	30.66	26.42	92.04	86.0	926.14	–	32,400	237.42	–	Chugh et al. (2016)
*A. niger* APS	Surface	IU/ml	3.84	1.21	3.68	14.2	7.4	40.1	–	2760	41	3.3	This study

Biofilms pose a serious clinical, industrial as well as environmental challenge because of their recalcitrant nature. Therefore, it is crucial to either prevent biofilm formation or to eradicate/disperse preformed biofilms to prevent biofouling. In this context, the EPS of the biofilm can be targeted and looking at the heterogeneity of the EPS, we evaluated the ability of in-house produced enzyme cocktail produced by the isolated strain *A. niger* APS in biofilm removal. To study the effect of in-house produced enzymes on biofilm eradication, biofilms of different pathogens were developed on microtitre plate. Since the density of biofilm produced under in vitro conditions depends on medium composition and incubation period, these factors were standardised for the development of biofilms of selected pathogens. LB and BHI were found to support enhanced biofilm formation in *E. coli* and *S*. Typhi as compared to TSB. LB and BHI are nutrient rich media. Proteins rich in amino acids like leucine, proline, serine, and aspartate are present in BHI and these amino acids might play a role in the production of adhesions, necessary for adherence (Singh et al. 2017). Similarly, tryptone, yeast extract and dextrose present in LB make it a suitable medium for the growth of microorganisms. Further, sodium chloride present in LB helps in the maintenance of proper isotonic environment of the broth, which further supports the growth of bacteria. In case of *S. aureus*, higher biofilm formation was supported by TSB which contains glucose enzymatic digests of casein and soybean meal and is a rich source of amino acids and nitrogenous substances. *S. aureus* is unable to ferment sugars such as inositol/myoinositol present in BHI which has been reported to result in resistance in pH fall, which, in turn, may be needed for robust biofilm architecture (Singh et al. 2017).

The extracellular polymeric substances (EPS) exuded by the biofilms act as a cementing material of the biofilms that holds the bacteria together as a community in a biofilm (Limoli et al. 2017). The extracellular polysaccharides are the major component of the biofilms and have been reported to be composed of homo- and hetero-polysaccharides composed of monomers like glucose, fucose, mannose, galactose, fructose, mannuronic acid, glucoronic acid or pyruvate based complexes. The bonds between these monomeric saccharides give rise to a plethora of different polysaccharides like levans, polymannans, cellulose, dextrans, amylopectin and alginate (Flemming et al. 2007; Limoli et al. 2017). The enzymes in the cocktail were found to efficiently disrupt the biofilms of all the pathogens however, the enzyme cocktail was found to be more effective against the biofilms of Gram-negative pathogens as the percentage reduction was found to be higher as compared to the Gram-positive counterparts. This could be due to the hydrolysis of the capsular polysaccharides (produced by both *Salmonella enterica* serovar Typhi and *E. coli*) by the action of multiple carbohydrases present in the cocktail. Various carbohydrases present in the cocktail might have degraded the polysaccharides present in the EPS leading to the loss of integrity of the EPS and finally resulted in the disruption of the biofilms which corroborated well with the morphological alterations observed in FESEM, wherein significant disruption of the biofilms was seen.

The significant clearance of the pre-formed slime layer on KDP might be due to the degradation of various polysaccharides present in the black gunk, as well as the ones produced by the microorganisms present in the biofilms, thus resulting in decreased viscosity and dispersion of the viscous slime layer. The clearance of the thick slime layer from KDP provides a promising insight for cleaning of drainage pipes, industrial pipelines and even sewerage systems. The clearance of slime formed on KDP is an interesting observation just giving the proof of concept. Only after executing studies pertaining to the concentration of enzyme cocktail and its appropriate formulation, the efficacy of enzyme cocktail can be compared with the commercially available agents. It may be noted that at this stage, it is not being claimed to be better than the commercially available agents but it may be another eco-friendly option to the chemical agents. Thus, the application of enzymes for the dispersal of biofilms and cleaning of slime would provide an interesting anti-biofouling alternative, especially in situations when the classical treatments involving chemical agents do not give satisfactory results.

Having an inclination towards process development using green technology, we have earlier reported the production of enzymatic cocktails from fungi (isolated from natural surroundings) followed by their application in diverse fields (Bansal et al. 2012; Janveja et al. 2013; Chugh et al. 2016; Rastogi et al. 2016). However, this is the first study wherein we have *reported the concurrent production of a cocktail of nine different carbohydrases including alginate lyase from yet another natural variant of Aspergillus niger pertinent* (i) *for the successful dispersal of biofilms consisting of heterogeneous matrix*, and (ii) *for the removal of viscous slime layer from drainage pipe formed under natural conditions*. This application holds a bright avenue to take care of manual scavenging which was banned in India in 1993 under the passing of the ‘Employment of Manual Scavengers and Construction of Dry Latrines (Prohibition) Act, 1993’. The law was extended and clarified to include insanitary latrines, ditches and pits in 2013 in India under the ‘Prohibition of Employment as Manual Scavengers and their Rehabilitation Act, 2013’. The application of enzymes for cleaning drainage pipes might help to achieve this target in toto.

## Supplementary information

**Additional file 1: Fig. S1.** Time course study for co-production of different enzyme components a) Cellulases; b) Hemicellulases; c) Pectinase and alginate lyase; d) Amylases; over a period of 10 days (240 h) under submerged fermentation. **Fig. S2.** Standardization of biofilm formation a) Effect of different media (Luria broth LB, Brain Heart Infusion Broth BHI, Tryptic Soy broth TSB) on biofilm formation b) Effect of incubation period.

## Data Availability

The authors confirm that the data supporting the conclusions is given within the article. Raw data is available with the corresponding authors and would be available on request.

## Abbreviations

ATCC: American Type Culture Collection
BLAST: Basic local alignment search tool
Bp: Base pair
CMCase: Carboxymethylcellulase
CSL: Corn steep liquor
EPS: Extracellular polymeric substance
FESEM: Field emission scanning electron microscopy
FPase: Filter paperase
ITS: Internal transcribed spacer
IU: International units
KDP: Kitchen drainage pipe
MTCC: Microbial Type Culture Collection
NCBI: National Center for Biotechnology Information
OVAT: One variable at a time
PBS: Phosphate Buffered Saline
PDA: Potato dextrose agar
SmF: Submerged fermentation
SSF: Solid state fermentation
rpm: revolutions per minute
