# Supplementary material for: A novel multi-enzyme preparation produced from Aspergillus niger using biodegradable waste: a possible option to combat heterogeneous biofilms
Source: AMB Express. 2020 Feb 21;10:36. doi: 10.1186/s13568-020-00970-3 (PMC7035411; doi:10.1186/s13568-020-00970-3)
Supplement: Supplementary file 1 — Additional file 1: Fig. S1. Time course study for co-production of different enzyme components a) Cellulases; b) Hemicellulases; c) Pectinase and alginate lyase; d) Amylases; over a period of 10 days (240 h) under submerged fermentation. Fig. S2. Standardization of biofilm formation a) Effect of different media (Luria broth LB, Brain Heart Infusion Broth BHI, Tryptic Soy broth TSB) on biofilm formation b) Effect of incubation period. [file 13568_2020_970_MOESM1_ESM.pdf]

(JOURNAL: AMB EXPRESS)

A novel multi-enzyme preparation produced from *Aspergillus niger* using biodegradable waste: a possible option to combat heterogeneous biofilms

Arashdeep Kaur[1], Valbha Rishi[2], Sanjeev Kumar Soni[1]*, Praveen Rishi[1]*

[1] Department of Microbiology, Panjab University, Chandigarh, India.

[2] Ex-PG Student, Department of Civil Engineering, National Institute of Technical Teachers' Training and Research, Chandigarh, India.

***Corresponding Authors:**

Prof. S. K. Soni
Department of Microbiology,
Panjab University,
Chandigarh, India.
Email: sonisk@pu.ac.in
Contact No. +91 9417351062
ORCID ID: https://orcid.org/0000-0002-1999-7061

Prof. Praveen Rishi
Department of Microbiology
Panjab University
Chandigarh, India.
E mail: rishipraveen@yahoo.com
Contact No. +919888895206
ORCID ID: https://orcid.org/0000-0001-5425-8064

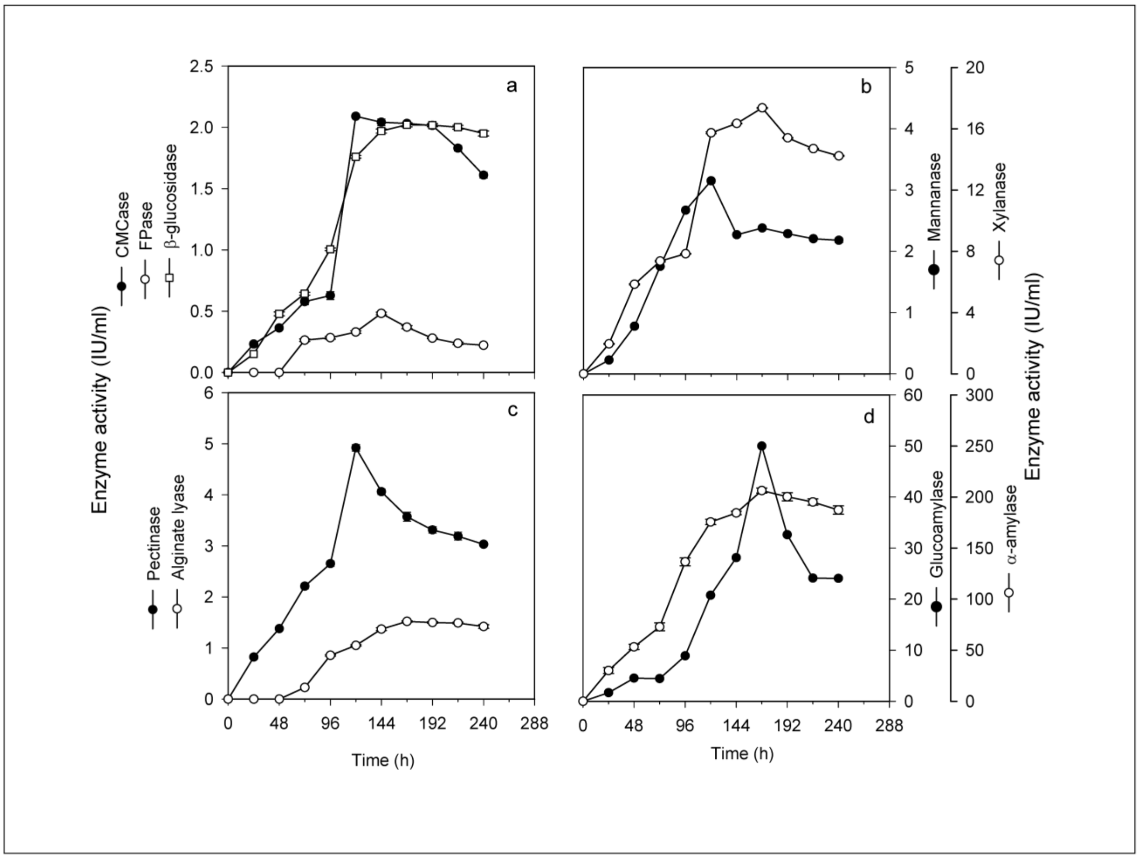

Additional file 1: Fig. S1 Time course for co-production of different enzyme components a) Cellulases; b) Hemicellulases; c) Pectinase and alginate lyase; d) Amylases; over a period of 10 days (240 hrs) under submerged fermentation.

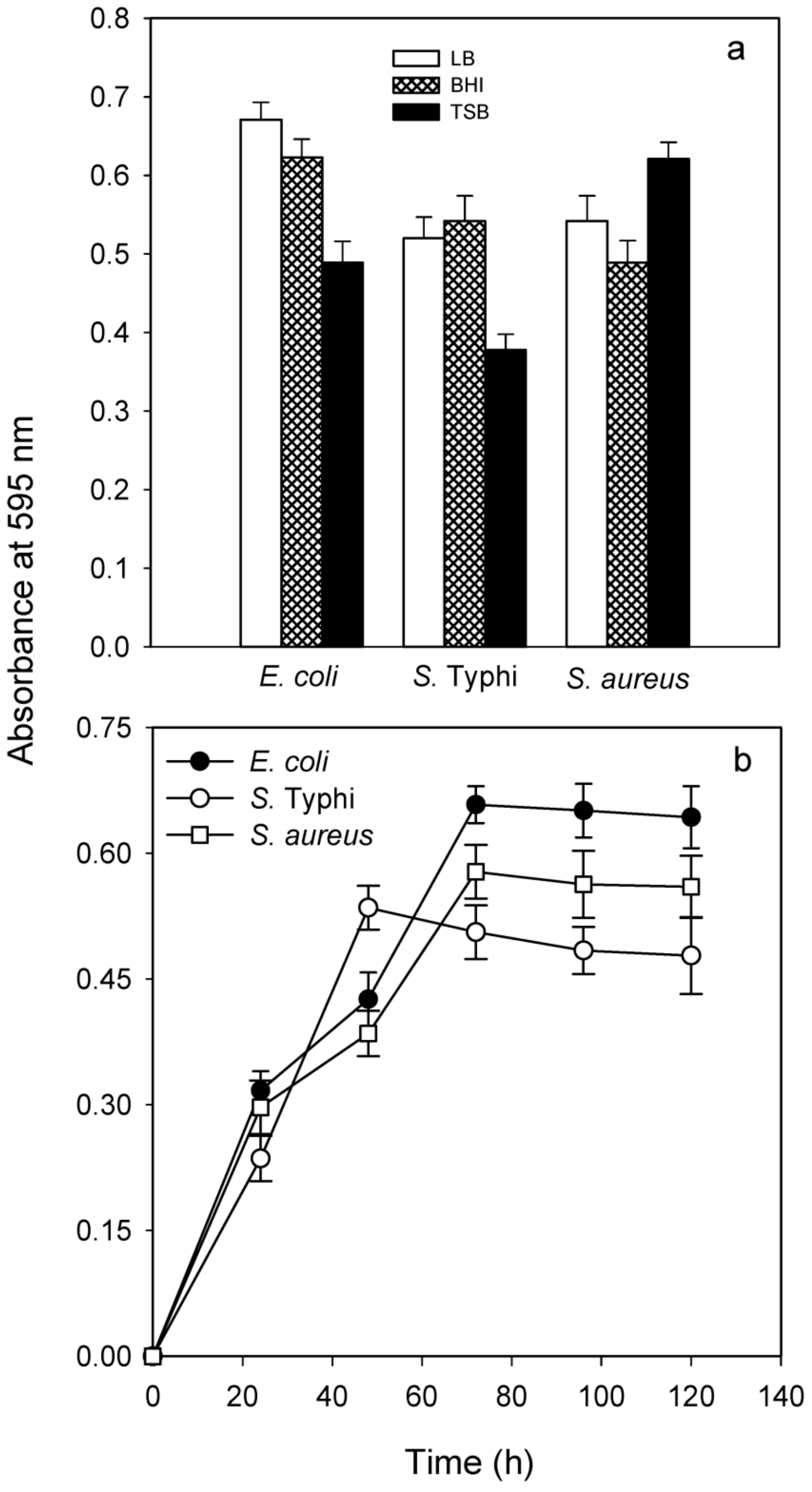

Additional file 1: Fig. S2 Standardization of biofilm formation a) Effect of different media (Luria broth LB, Brain Heart Infusion Broth BHI, Tryptic Soy broth TSB) on biofilm formation b) Effect of incubation period.
